# Vitamin D as a Principal Factor in Mediating Rheumatoid Arthritis-Derived Immune Response

**DOI:** 10.1155/2019/3494937

**Published:** 2019-05-07

**Authors:** Muhammad M. Aslam, Peter John, Attya Bhatti, Sidrah Jahangir, M. I. Kamboh

**Affiliations:** ^1^Atta-ur-Rahman School of Applied Biosciences, National University of Sciences and Technology, Islamabad, Pakistan; ^2^Department of Human Genetics, Graduate School of Public Health, University of Pittsburgh, Pittsburgh, PA, USA

## Abstract

Rheumatoid arthritis (RA) is a systemic multifactorial autoimmune disorder. The interactions between diverse environmental and genetic factors lead to the onset of this complex autoimmune disorder. Serum levels of vitamin D (VD) are involved in the regulation of various immune responses. Vitamin D is a key signaling molecule in the human body that maintains calcium as well as phosphate homeostasis. It also regulates the functions of the immune system and, thus, can play a substantial role in the etiology of various autoimmune disorders, including RA. Low serum VD levels have been found to be associated with a higher risk of RA, although this finding has not been replicated consistently. The molecular mechanisms by which VD influences autoimmunity need to be further explored to understand how variation in plasma VD levels could affect the pathogenesis of RA. This mini-review focuses on the influence of VD and its serum levels on RA susceptibility, RA-associated complexities, treatment, and transcriptome products of key proinflammatory cytokines, along with other cytokines that are key regulators of inflammation in rheumatoid joints.

## 1. Rheumatoid Arthritis

Rheumatoid arthritis (RA) is a systemic autoimmune multifactorial complex disease [1]. The key characteristic of this complex autoimmune disorder is the inflammation of the small joints [2–4]. Rheumatoid arthritis is associated with significant morbidity and mortality. The worldwide prevalence of RA is one percent [5]. The disease is usually more common among females than males [6, 7]. Mortality data from the United Nations Population Prospects database from 1987-2011 and World Health Organization mortality database for 31 countries show that RA accounted for almost 18 percent of all deaths caused by different forms of arthritis and other musculoskeletal disorders [8].

The interface between diverse environmental and genetic elements leads to the onset of RA [9]. The initial stages of RA are usually not evident clinically. One of the disease hallmarks of RA is the production of rheumatoid factor (RF) triggered by the autoimmunity. The imbalance of different immunological mediators leads to cellular damage, which in the case of RA manifests in bone and joint damage [10]. Cytokines are an imperative regulatory element in the pathogenesis of RA. Generally, the cytokines involved in RA can be grouped into two main categories: proinflammatory and anti-inflammatory cytokines.

Tumor necrosis factor alpha (TNF*α*), interleukin1 (IL-1), interleukin6 (IL-6), and interleukin17 (IL-17) are key proinflammatory cytokines that play vital regulatory roles in the chronic inflammation of joints and associated cartilage and in bone deformation. TNF*α* is an inflammatory mediator that is arthritogenic even in its membrane-bound form [11]. IL-1 is a central cytokine in both RA and RA-mediated destruction of cartilage. IL-6 contributes to the production of autoantibodies. IL-6 also regulates the activation and differentiation of various immune cells. These cytokines have been targeted for gaining therapeutic insights into RA [12]. Proinflammatory cytokines have a significant role in the disease occurrence and severity of RA. Multiple genetic studies focusing on key proinflammatory cytokine genes have investigated the role of common genetic variation in relation to RA risk, disease severity index, and drug response. Polymorphisms in the regulatory regions of these cytokine genes can significantly affect the binding of various transcription factors that can influence the risk of RA [13–17]. Since the focus of this short review is on the effect of VD on proinflammatory cytokines, anti-inflammatory cytokines are not discussed here.

## 2. Vitamin D (1,25-Dihydroxyvitamin D)

**Figure 1:**
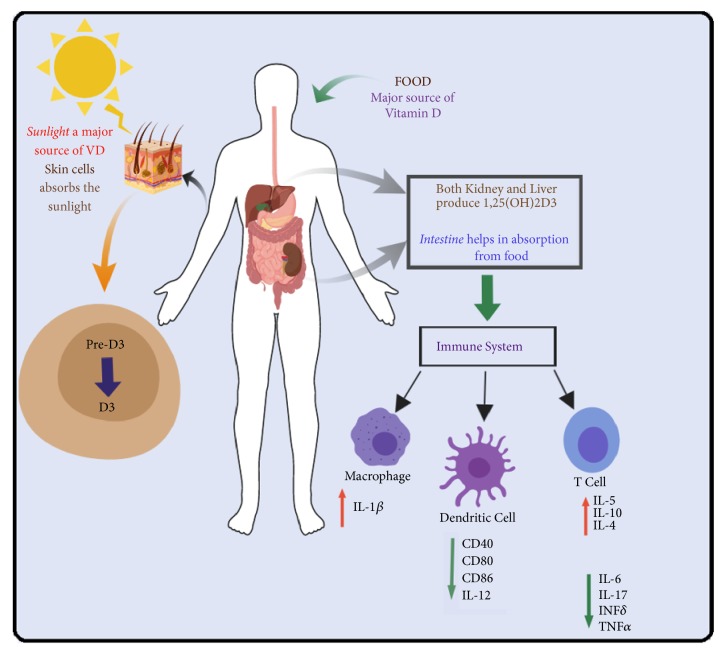
Route map of vitamin D from production to action.

Vitamin D (VD) is a secosteroid hormone that is produced mainly by skin under the exposure of *β*-radiations and UV light [18]. Kidney and liver are major players for VD metabolism [19, 20]. It can also be supplemented through diet where gastrointestinal absorption takes it to blood circulation [21]. VD is considered as one of the essential nutrients in the human body. Its most significant role is to maintain calcium and phosphate homeostasis. Optimal serum VD level is 30 ng/ml [22, 23]. Different forms of VD have different activity levels [24]. Once it is generated in the body through sunlight or after body received it from food, VD is chemically converted to its active form (Figure 1). Two different enzymes generate the active form of VD. First, 25-hydroxylase, a liver enzyme, converts recently produced inactive VD to 25-hydroxyvitamin D [25(OH)D] that subsequently is activated by a kidney enzyme, 1*α*-hydroxylase, to form 1,25-dihydroxyvitamin D [1,25(OH)_2_D3] [25]. Activated VD is responsible for maintaining calcium and phosphate homeostasis by increasing intestinal phosphate and calcium absorption. VD plays an essential role in several physiological processes, including bone formation, immunity, cellular growth, and cellular differentiation [26]. Serum VD level variation has been implicated in various diseases, including cancer, metabolic syndrome, immune system disorders, frailty, cardiovascular disorders, and neurological disorders [27–31]. A microarray analysis has estimated that VD regulates 5% of the human genome either directly or indirectly and regulates the physiological behavior of more than 36 different cell types [32]. Many small scale genetic and genome-wide association studies (GWAS) have implicated multiple genetic loci (*GC, DHCR7, CYP2R1, CYP24A1, SEC23A, AMDHD1, A2BP1, GPR114, DAB1, MLL3, FOXA2, *and* HMCN1*) that are involved in the synthesis, transportation, metabolism, and degradation of VD [33].

Vitamin D receptor (VDR) is a member of the nuclear hormone receptors' family [34]. VD acts as a ligand for VDR. The lipophilic 1,25(OH)_2_D_3_ can easily pass through cellular membranes and binds to its receptors without the involvement of any additional signal transduction steps, as is the case of the ligand molecules that bind to transmembrane receptors [35]. Since VDR is ubiquitously expressed, a wide range of different cell types are responsive to VD [36]. VDR is expressed in chondrocytes and synoviocytes present in inflamed joints of RA subjects. Genetic variation in the* VDR* gene has been linked to RA risk [37–40].

## 3. VD and Immunity

The discovery of the existence of VDR on peripheral blood mononuclear cells (PBMCs) and its role in the pathogenesis of RA laid down the foundation about the potentially important role of VD as an important immunity regulator [41–43]. VD plays a vital part in the regulation of various immunity mediated responses [44]. It has a significant role in controlling innate and adaptive immunity but in an antagonistic manner [45]. VD controls the innate and adaptive immune systems mainly through toll-like receptors (TLRs) and differentiation of T-cells, predominantly Th17 cells, and these Th17 cells have a crucial role in RA pathology [46]. VD modulates the regulation and differentiation of immune cells. It controls the production and secretion of autoantibody in B-cells [47]. It suppresses the proliferation and differentiation of B-cells by inducing apoptosis in activated B-cells [48]. VD obstructs the T-cells proliferation and inhibits the synthesis of IL2, INF-*γ*, and TNF*α* cytokines [49].

## 4. VD and Autoimmunity

In an autoimmune response, VD is involved in maintaining an optimum balance between Th1 and Th2 to suppress the autoimmune response mediated by T cells, by regulating CD4^+^T cells production and activity [43]. It also halts antigen representation [50]. To overcome the effects of autoreactive T cells, VD increases the regulatory T cells activity [51]. Estrogen in RA synovial tissue boosts the immune response and VD is found to downregulate the estrogen synthetase activity, hence controlling the autoimmune response [52]. VD has an immunosuppressive effect and the physiologic concentration of VD has been shown to provide protection against autoimmune diseases [53, 54]. Changes in serum availability of VD can affect various cells and their normal signaling cascades. This can lead to disturbances in homeostasis at the molecular level, leading to onset and pathogenesis of various disorders, especially those related to calcium and bone metabolism and immune system dysfunction. Deficiency of VD has been linked to many autoimmune disorders, including insulin-dependent diabetes mellitus, systemic lupus erythematosus (SLE), and RA [55–57].

## 5. Vitamin D and Tumor Necrosis Factor-Alpha (TNF*α*)

Inflammation in RA occurs due to the abundant presence of inflammation-promoting cytokines [58]. TNF*α* is implicated in systemic inflammation. This is mainly synthesized by activated macrophages. Numerous other cell types can also produce TNF*α*, including fibroblast, monocytes, natural killer cells, and mast cells [59]. Most of these TNF*α* producing cells have VDR [60, 61]. TNF*α* is encoded by the* TNFA* gene that is present on chromosome 6p21.3. The gene is ~3 kb and comprises 4 exons [62]. TNF*α* promotes inflammatory signaling and performs a key role in the onset and pathogenesis of RA. The level of TNF*α* has been shown to be higher in RA patients than controls, as TNF*α* is involved in inflammation followed by joint destruction [63]. However, the role of* TNFA* genetic variations in RA has not been established yet [64].

Studies intending to explore the effect of VD treatment on TNF*α* production have shown an inverse correlation between these two. This correlation has been investigated by quantification of mRNA or level of protein production and protein release in numerous studies. In PBMCs,* TNFA* transcriptome, as well as proteome, was reported to be inversely correlated with VD stimulation [65]. A VDR binding sequence has been found in the promoter of* TNFA*. VD levels can affect the binding of VDR to its target sequences in the upstream regulatory regions of the* TNFA* gene, which in turn can regulate the transcription of* TNFA* mRNA. VD levels, however, are not linked with* TNFA* mRNA stability. VD, therefore, regulates TNF*α* at transcriptional level [66]. A study conducted on a mouse model concluded that VD acts as a shield against RA because this promotes the apoptosis of fibroblast-like synoviocytes, which are key factors for cartilage destruction in RA [67]. Another study conducted on healthy women showed an inverse correlation between VD and TNF*α* concentration and suggested the preventive role of VD against inflammatory conditions [68].

## 6. Vitamin D and Interleukin-1 (IL-1)

IL-1 family is a group of 11 different cytokines [69]. Interleukin 1 alpha (IL-1*α*) and interleukin 1 beta (IL-1*β*) are the most studied members of this immunoregulatory molecule family. These cytokines are encoded by IL1A and IL1B genes that are located on 2q14. These two cytokines have a common antagonist called IL-1 receptor antagonist (IL-1Ra). The receptor for IL-1*α* and IL-1*β* is IL-1 receptor I (IL-1RI). IL-1Ra also binds to IL-1RI but it cannot induct any intracellular signaling and thus it acts to regulate the action of IL-1*α* and IL-1*β* [70]. IL-1*β* is produced by endothelial cells, monocytes, macrophages, activated T cells, and B cells [71]. It is expressed in mononuclear blood cells and synovial membrane [72]. It is involved in proteoglycan degradation and inhibits the synthesis of proteoglycan [73]. IL-1*β* has a key role in articular damage in RA and it also elicits the production of other cytokines, especially IL-6, in RA [74]. Studies of RA in animal models have shown the involvement of IL-1*α* and IL-1*β* in joint damage and cartilage degradation [75, 76].

IL-1*β* is found in infected cells and VD elevates IL-1*β* levels in macrophages during infection through direct transcription mechanism [77]. Similarly, another study showed that VD induced IL-1*β* production in lipopolysaccharide-treated human monocytes-derived macrophages and it also increased the production and phosphorylation of IL-1*β* transcriptional regulatory factor (C/EBP*β*-CCAAT enhancer binding protein *β*) [78]. Another study conducted to find out the effect of VD on levels of proinflammatory cytokines found that VD significantly downregulated the levels of IL-1*β* [79]. VD has been reported to be inversely associated with IL-1*α* and IL-1*β* levels [80, 81], although a few earlier studies reported a positive correlation of VD and IL-1*α* and IL-1*β* [82–84]. Similar to IL-6 production, the levels and kind of influence VD has on IL-1 transcriptome depends on several additional factors. In human monocytic cell lines, the presence or absence of any connection between VD levels and IL-1 expression depends on the presence/absence and the nature of costimulus being present [85].

## 7. Vitamin D and Interleukin-6 (IL-6)

IL-6 is a monomeric glycoprotein of 26 kDa that is encoded by an interleukin-6 gene (*IL6*) located on 7p21. The glycoprotein is arranged into four long helical chains [86, 87]. IL-6 is a pleiotropic cytokine that is released by a range of different immune cells, including epithelial cells, fibroblasts, monocytes, and T cells [88]. The IL-6 receptor consists of two different polypeptide chains: gp130 and IL-6 receptor (IL-6R) while IL-6R specifically binds to gp130 and it serves to mediate intracellular signaling that can be either via JAK (Janus kinase)/STAT(signal transducer and activator of transcription) pathway or via mitogen-activated protein (MAP) kinase pathway [89, 90]. The STAT/JAK intracellular signaling pathway is known to play a vital role in immune-related responses that are mediated by IL-6 [91]. IL-6 is a primary mediator of inflammation. The levels of this cytokine are considerably elevated in the serum of RA patients [92–94]. IL-6 has been known to contribute towards the production of autoantibodies and it also acts as a regulator of TH-cells differentiation [95]. The signaling pathway triggered by IL-6 ultimately leads to joint inflammation and bone erosion in RA [96]. IL-6 is also involved in the initiation of the acute-phase response, the proliferation of synovial fibroblasts, and the stimulation of the precursor cells of hematopoietic lineage [97].

Serum levels of VD have been reported to be inversely related to serum IL-6 levels [98]. VD has been implicated as a downregulator of IL-6 mRNA levels in prostate cells. VD inhibits p38 molecule by the induction of MAPK phosphatase-1 (MKP1). This leads to the dephosphorylation of p38 by MKP1 and thus the activated p38 levels are reduced. p38 inhibition, in turn, is responsible for the reduction of IL-6 transcripts in the target cells [99]. IL-6 expression regulation has also been correlated with the differentiation of immune cells. The expression of IL-6 has been, therefore, linked with the degree of maturation of the immune cell, cytokine, and other signaling molecules [85]. Th17 cells are considered a crucial component of autoimmune-mediated response and 1,25(OH)_2_D_3_ has shown to stop the IL-6 expression, which in turn stimulate the production of Th17 cells [100, 101]. Exposure of VD reduces IL-6 levels in TNF- *α* stimulated synovial stroma cells (SSCs) from RA patients [102].

## 8. Vitamin D and Interleukin-17 (IL-17)

IL-17 is an inflammatory cytokine which is produced mainly by Th17 and other innate immune cells that have a crucial role in immune response and tissue impairment in RA [103]. It is mostly expressed in synovial fluid and synovium of RA patients [104]. Due to the immunomodulatory effect of VD on Th17 cells, it was found that active form of VD decreases the production of Th17 from CD4+T cells in humans and also it cuts down the expression of IL-17 in CD4+ T cells [105]. A recent study provides support to this observation where deficiency of VD in RA patients was found to affect Th17 cells function and, hence, IL-17 production, indicating that sufficient levels of VD may guard RA patients against IL-17 mediated immune response [106]. Some animal model studies have also reported similar findings where VD was associated with reduced production of IL-17 [107, 108]. T cells, especially Th17, are one of the main target sites for VD. VD action on T-cells halts the T-helper cells cytokines and alters the cytokine expression pattern of antigen presenting cells [109–112].

## 9. Vitamin D and Other Cytokines

Being an autocrine growth factor, IL-2 plays a significant role in optimum immune system functioning by acting as an activator, growth factor, and key component for T-cells differentiation [113, 114]. In the adaptive immune system, multiple T lymphocytes are favorite action sites for VD. VD is found to be an inhibitory factor for Th1 cells and subsequently reduced the production of INF*γ* and IL-2, which are important Th1 cytokines [115, 116]. In an* in vitro* study, it was found that VD regulated the Th2 production and Th2 cells were the main source of IL-2 and IL-10 production. Th2 cells are also involved in Th1 cells function inhibition [117]. A study conducted on human T-cell line confirmed that VD suppressed the IL-2 gene expression and reduced the IL-2 production by blocking the positive regulatory elements of transcriptional factor (NFAT) within the promoter region of the IL-2 gene [118]. In most of the cases, VD is found to downregulate the production of different cytokines, but, in case of IL-4 and IL-10, VD has an opposite effect where it upregulates the synthesis of IL-4 [119] and IL-10 [120]. An* in vitro* study showed that treatment of 1,25(OH)_2_D_3_ on CD4+Mel14+ T cells enhanced the synthesis of Th2 lymphocytes and ultimately increased the production of IL-4, IL-5, and IL-10 [121]. IL-12 determines the fate of T cells and its levels are found to be higher in RA patients [122]. In human PBMCs, VD was found to have an inhibitory effect on the production of IL-2 and IL-12 [123]. VD also blocks the differentiation of a dendritic cell and thus inhibits the IL-12 production. The complex of 1,25(OH)2D3, VDR, and NF*κ*B hinder with NF*κ*B-derived transcription of IL-12 [124]. VD also downregulates the production of IL-12 and IL-23 by elevating the production of IL-10 [125, 126].

## 10. Connection between VD and RA

Vitamin D has been shown to act as a key player in the onset and pathogenesis of RA. In murine RA, the hormonally activated form of VD (1,25-Dihydroxvitamin D3 [1,25(OH)2D3]) has been implicated in preventing the onset and RA pathogenesis [119].* In vitro* studies in different cell lines that mimic RA like pathology have revealed that VD promotes anti-inflammatory response [127]. An* In vivo *study on a transgenic mouse model of RA showed that deletion of VDR was associated with inflammation followed by bone loss [128].

The prevalence of RA has been found to decrease in individuals with high intake of VD, including both dietary and supplemental forms of VD [129]. Epidemiological data have revealed that a significant number of RA patients (30-63%) have decreased VD levels [130]. VD intake is inversely associated with RA activity [131]. Distribution of serum VD levels has been examined in a number of RA case-control studies. A vast majority of these studies have found significantly different VD levels between cases and controls and these results are summarized in Table 1. Below we summarize the outcomes of significant studies.

A study conducted on RA patients that were not taking any VD supplements found a severe deficiency of VD [132]. A recent meta-analysis which combined data from fifteen different studies on a total of 1,143 RA patients and 963 controls reported the same inverse correlation between serum VD levels and disease severity [133]. A similar association between disease activity score (DAS28) and serum VD levels was found [134]. A cross-sectional study measured serum VD levels and reported VD insufficiency in a group of rheumatic patients [135]. Another study conducted on Caucasian women also reported serum VD insufficiency in RA patients as compared to controls [136]. A few other studies also reported a similar inverse association between VD levels and disease severity [137–141]. A recent meta-analysis combined results from different reports on 2,148 cases and 1,991 healthy controls, reported lower serum VD levels in RA patients as compared to healthy controls, and further reported an inverse correlation between serum VD levels and disease severity score [142]. Wang et al. [143] studied the effect of serum VD levels on 154 RA patients and reported an inverse relationship between VD levels and disease activity and anti-CCP level. A European League Against Rheumatism (EULAR) that supported a study on 625 RA patients and 276 healthy controls from 13 different European countries also reported hypovitaminosis in RA patients and inversely correlated serum VD levels with RA-associated complexities [144]. A study on a much larger sample size of 894 RA and 861 healthy controls reported an inverse correlation between serum VD levels and RA disease activity [145]. Another study on 93 RA patients and 31 healthy controls from an Iranian population also reported the inverse association between serum VD levels and RA severity and suggested VD supplementation for RA treatment along with other regular medications [146]. A study conducted on the Turkish population reported an inverse relationship between serum VD levels and RA susceptibility but did not find any association between serum VD levels and disease activity [147]. Similar results have been published by research published on Iranian population [148].

Severe deficiency of VD has been reported in early inflammatory arthritis [149]. A study conducted on 4,793 Japanese RA patients reported a severe deficiency of VD in RA patients and indicated an inverse association between levels of VD and RA related clinical symptoms [150]. Similarly, another study conducted on European RA patients reported the same results and linked VD levels inversely with RA-associated clinical symptoms, but it did not demonstrate any correlation between serum VD levels and disease severity score [151]. Studies conducted on the Italian population also reported VD deficiency in RA patients [152, 153]. In line with these results, data from North Italy rheumatology outpatients' clinic demonstrated 87% prevalence of VD deficiency in patients suffering from autoimmune rheumatic diseases [154]. Parallel to these results, almost 90% of hypovitaminosis D was reported in RA patients from the UK and Swiss outpatients clinics [155, 156]. Comorbidities in Rheumatoid Arthritis (COMORA) cohort comprising 1,431 patients from 15 different countries also found low serum VD levels with RA incidence and comorbidities [157]. A study conducted on Saudi Arabian RA patients reported VD as a good predictor of disease activity [158].

In RA treatment, combination therapy of denosumab and VD increases bilateral total hips bone mineral density (H-BMD) [159]. Another study suggested the role of VD in maintaining endothelial homeostasis in RA patients based on VD levels and CD34+ cell count in RA patients [160]. Two more studies suggested the potential immunomodulatory role of VD that can have a promising effect in RA patients [161, 162]. VD also affects other disease parameters, including Th17 cell count and incidence of anti-CCP antibodies [163]. Despite the immunomodulatory properties of VD, the beneficial role of VD supplementation as a component of RA treatment has produced inconsistent results [164–166].

## 11. VD and RA Related Complexities

A recent study in Northwest China found that RA patients with depression have much lower serum VD levels (mean= 15.24 ± 8.78 ng/mL) as compared to RA patients without depression (mean= 24.68 ± 10.98 ng/mL) and associated hypovitaminosis with depression, anxiety, and disease activity in RA patients [167]. Another study also associated low serum VD levels with increased neuropathic pain in RA patients [168]. Furthermore, low serum VD levels are inversely associated with ROS (reactive oxygen species) levels in RA patients [169]. A recent data indicate that low serum VD levels in RA patients may lead to secondary osteoporosis [170].

## 12. Conclusions

The human body can synthesize VD under the exposure of *β*-radiations and UV light or can absorb it through food. Kidney and liver metabolize the absorbed VD. VD maintains the calcium and phosphate homeostasis in the body. VD can regulate innate and adaptive immunity mainly through B and T-cell production and differentiation. It inhibits the synthesis of IL2, INF-*γ*, and TNF*α*. Immunomodulatory properties of VD have made it an important factor in multiple autoimmune conditions. VD serum levels are inversely associated with RA susceptibility, disease activity, and related pathological complexities. VD is a significant regulator of various genes involved in the immune system and plays an important role in various immune-related responses, including the expression of proinflammatory cytokines. VD, through suppression of cytokines levels, can prevent the onset and pathogenesis of RA. Therefore, VD deficiency, coupled with genetic and environmental factors, may lead to the onset of RA. Additional studies are needed to explore the precise molecular pathways and mechanisms by which VD levels mediate RA-derived immune response. Research on the potential role of VD supplementation in RA treatment has produced inconsistent results; additional large-scale pharmacological research is required to find out the effect of VD augmentation during the treatment of RA.

## Figures and Tables

**Table 1:** Summary of the relationship between serum VD levels and RA in different populations.

Character	Association	Population	Sample size	Reference
Serum VD levels and RA	Inverse	Poland	97 cases, 28 controls	[132]

Serum VD levels and RA	Inverse	Meta-analysis	1,143 cases, 963 controls	[133]

Serum VD levels and RA	Inverse	South European	120 cases, 65 controls	[134]

Serum VD levels and RA	Inverse	Croatia	53 RA patients	[135]

Serum VD levels and RA	Inverse	Caucasian (Argentina)	42 cases, 48 controls	[136]

Serum VD levels and RA	Inverse	India	80 cases, 80 controls	[137]

Serum VD levels and RA, IL-17/IL-23, and bone loss	Inverse	Chinese	130 cases, 80 controls	[138]

Serum VD levels and RA	Inverse	Egypt	63 cases, 62 controls	[139]

Serum VD levels and RA	Inverse	Saudi Arabia	55 cases, 40 controls	[140]

Serum VD levels and RA and musculoskeletal pain	Inverse	Greece	44 cases, 44 controls	[141]

Serum VD levels and RA	Inverse	Meta-analysis	3,489 RA patients	[142]

Serum VD levels and RA, anti-CCP antibody	Inverse	Chinese	154 cases, 60 controls	[143]

Serum VD levels and RA & associated complexities	Inverse	13 European countries	625 cases, 276 controls	[144]

Serum VD levels and disease severity	Inverse	Iran	91 cases, 31 controls	[146]

Serum VD levels and RA	Inverse	Japan	4,793 RA patients	[150]

Serum VD levels and RA	Inverse	Italy	1,191 cases, 1,019 controls	[152]

Serum VD levels and RA	Inverse	Italy	1,168 RA patients	[153]

Serum VD levels and RA associated depression and anxiety	Inverse	Northwest China	161 RA patients	[167]

Serum VD levels and neuropathic pain in RA patients	Inverse	Turkey	93 RA patients	[168]

Serum VD levels and RA	Inverse	COMORA cohort (15 countries)	1431 RA patients	[157]

Serum VD levels and ROS in RA patients	Inverse	India	100 cases, 50 controls	[169]

Combination therapy of VD + denosumab and H-BMD	Positive	Japan	22 monotherapy, 21 combination therapy	[159]

(i) Serum VD levels and RA (ii) Serum VD levels and disease activity	(i) Inverse (ii) No association	Turkey	55 cases, 45 controls	[147]

(i) Serum VD levels and disease activity (ii) Serum VD levels and RA	(i) No association (ii) Inverse	Iran	99 cases, 68 controls	[148]
